# Tris(hy­droxy­meth­yl)methanaminium trifluoro­acetate

**DOI:** 10.1107/S1600536811052226

**Published:** 2011-12-10

**Authors:** Ming-Liang Liu

**Affiliations:** a Ordered Matter Science Research Center, Southeast University, Nanjing 211189, People’s Republic of China

## Abstract

In the crystal structure of the title salt, C_4_H_12_NO_3_^+^·C_2_F_3_O_2_^−^, N—H⋯O and O—H⋯O hydrogen bonds link the ions, forming a complex three-dimensional network.

## Related literature

For background to ferroelectric complexes, see: Fu *et al.* (2011 ▶); Zhang *et al.* (2010 ▶). For a related structure, see: Rudman *et al.* (1983 ▶).
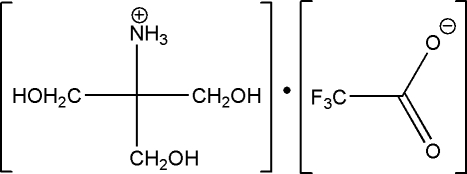


## Experimental

### 

#### Crystal data

C_4_H_12_NO_3_^+^·C_2_F_3_O_2_^−^
*M* *_r_* = 235.17
Monoclinic, 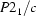

*a* = 8.5137 (17) Å
*b* = 6.1210 (12) Å
*c* = 18.283 (4) Å
β = 99.34 (3)°
*V* = 940.1 (3) Å^3^
*Z* = 4
Mo *K*α radiation
μ = 0.18 mm^−1^
*T* = 293 K
0.36 × 0.32 × 0.28 mm

#### Data collection

Rigaku Mercury2 diffractometer
Absorption correction: multi-scan (*CrystalClear*; Rigaku, 2005 ▶) *T*_min_ = 0.963, *T*_max_ = 0.971
9320 measured reflections
2148 independent reflections
1755 reflections with *I* > 2σ(*I*)
*R*_int_ = 0.041
3 standard reflections every 180 reflections  intensity decay: none

#### Refinement

*R*[*F* ^2^ > 2σ(*F* ^2^)] = 0.061
*wR*(*F* ^2^) = 0.155
*S* = 1.02
2148 reflections
137 parameters
H-atom parameters constrained
Δρ_max_ = 0.62 e Å^−3^
Δρ_min_ = −0.57 e Å^−3^

### 

Data collection: *CrystalClear* (Rigaku, 2005 ▶); cell refinement: *CrystalClear*; data reduction: *CrystalClear*; program(s) used to solve structure: *SHELXS97* (Sheldrick, 2008 ▶); program(s) used to refine structure: *SHELXL97* (Sheldrick, 2008 ▶); molecular graphics: *SHELXTL* (Sheldrick, 2008 ▶); software used to prepare material for publication: *SHELXTL*.

## Supplementary Material

Crystal structure: contains datablock(s) I, global. DOI: 10.1107/S1600536811052226/go2038sup1.cif

Structure factors: contains datablock(s) I. DOI: 10.1107/S1600536811052226/go2038Isup2.hkl

Supplementary material file. DOI: 10.1107/S1600536811052226/go2038Isup3.cml

Additional supplementary materials:  crystallographic information; 3D view; checkCIF report

## Figures and Tables

**Table 1:** Hydrogen-bond geometry (Å, °)

*D*—H⋯*A*	*D*—H	H⋯*A*	*D*⋯*A*	*D*—H⋯*A*
O1—H1⋯O2^i^	0.82	1.86	2.644 (2)	159
O2—H2⋯O5	0.82	1.86	2.673 (3)	170
O3—H3⋯O4^ii^	0.82	1.87	2.677 (3)	170
N1—H1*A*⋯O4^iii^	0.89	1.91	2.795 (3)	171
N1—H1*B*⋯O1^iv^	0.89	1.98	2.854 (2)	168
N1—H1*C*⋯O3^v^	0.89	2.02	2.899 (2)	169

Symmetry codes: (i) 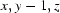; (ii) 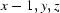; (iii) 
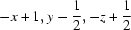
; (iv) 
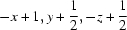
; (v) 
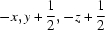
.

**Fig. 1.**
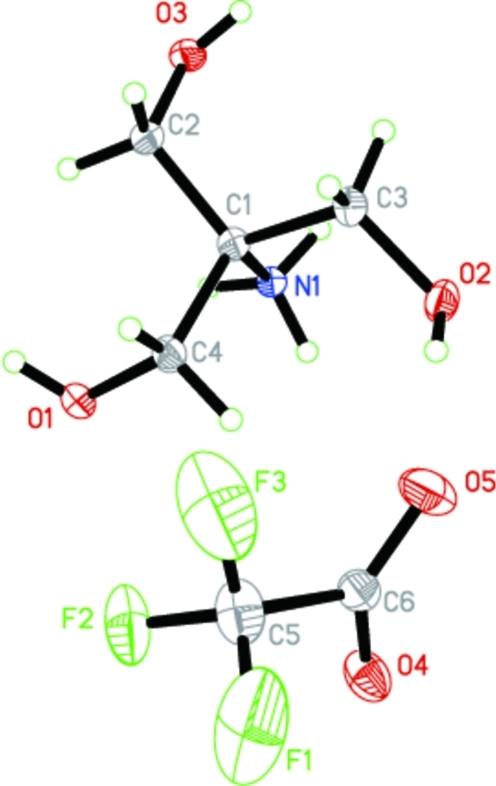
The molecular structure of the title compound, showing the atomic numbering scheme with 30% probability displacement ellipsoids.

**Fig. 2.**
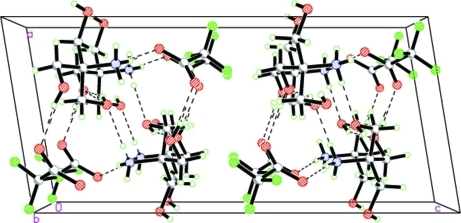
Crystal structure of the title compound with view along the b axis. Intermolecular interactions are shown as dashed lines.

## supplementary crystallographic information

### Comment

Recently much attention has been devoted to crystals containing organic ions and inorganic ions due to the possibility of tuning their special structural features and their potential ferroelectrics properties (Fu *et al.*, 2011; Zhang *et al.*, 2010.).

The compound (C_4_H_12_O_3_N)^+^(C_2_F_3_O_2_)^-^ has an asymmetric unit that consists of one tris(hydroxymethyl)methanaminium cation and one trifluoroacetate anion (Fig 1). N-H···O and O-H···O hydrogen bonds form a complex three-dimensional network, (Fig 2). The trifluoromethyl group is quite mobile, but examination of a difference map in the plane of the fluorine atoms does show that the fluorine atoms exist as three distinct atoms.

For structure of the related tris(hydroxymethyl)methanaminium hydrogenhalides seen (Rudman *et al.*, 1983).

### Experimental

1.21 g (0.01 mol) of tris(hydroxymethyl)methanaminium was firstly dissolved in 30 ml of ethanol, to which 1.14 g (0.01 mol) of trifluoroacetic acid was added at the ambient temperature. Single crystals suitable for X-ray structure analysis were obtained by the slow evaporation of the above solution after 3 days in air.

The dielectric constant of the compound as a function of temperature indicates that the permittivity is basically temperature-independent (*ε* = C/(T–T_0_)), suggesting that this compound is not ferroelectric or that there may be no distinct phase transition occurring within the measured temperatur (below the melting point).

### Refinement

H atoms were placed in calculated positions (N—H = 0.89Å; O—H = 0.82Å; C—H = 0.93Å for C*sp^2^* atoms and C—H = 0.96Å and 0.97Å for C*sp^3^* atoms), assigned fixed *U*_iso_ values [*U*_iso_ = 1.2*U*eq(C*sp^2^*) and 1.5*U*eq(C*sp^3^*, N and O )] and allowed to ride.

### Crystal data

**Table:**

C_4_H_12_NO_3_^+^·C_2_F_3_O_2_^−^	*F*(000) = 488
*M**_r_* = 235.17	*D*_x_ = 1.661 Mg m^−^^3^
Monoclinic, *P*2_1_/*c*	Mo *K*α radiation, λ = 0.71073 Å
Hall symbol: -P 2ybc	Cell parameters from 1755 reflections
*a* = 8.5137 (17) Å	θ = 3.4°
*b* = 6.1210 (12) Å	µ = 0.18 mm^−^^1^
*c* = 18.283 (4) Å	*T* = 293 K
β = 99.34 (3)°	Block, colourless
*V* = 940.1 (3) Å^3^	0.36 × 0.32 × 0.28 mm
*Z* = 4	

### Data collection

**Table:**

Rigaku Mercury2 diffractometer	1755 reflections with *I* > 2σ(*I*)
Radiation source: fine-focus sealed tube	*R*_int_ = 0.041
graphite	θ_max_ = 27.5°, θ_min_ = 3.5°
CCD_Profile_fitting scans	*h* = −11→11
Absorption correction: multi-scan (*CrystalClear*; Rigaku, 2005)	*k* = −7→7
*T*_min_ = 0.963, *T*_max_ = 0.971	*l* = −23→23
9320 measured reflections	3 standard reflections every 180 reflections
2148 independent reflections	intensity decay: none

### Refinement

**Table:**

Refinement on *F*^2^	Secondary atom site location: difference Fourier map
Least-squares matrix: full	Hydrogen site location: inferred from neighbouring sites
*R*[*F*^2^ > 2σ(*F*^2^)] = 0.061	H-atom parameters constrained
*wR*(*F*^2^) = 0.155	*w* = 1/[σ^2^(*F*_o_^2^) + (0.0616*P*)^2^ + 1.3289*P*] where *P* = (*F*_o_^2^ + 2*F*_c_^2^)/3
*S* = 1.02	(Δ/σ)_max_ < 0.001
2148 reflections	Δρ_max_ = 0.62 e Å^−^^3^
137 parameters	Δρ_min_ = −0.57 e Å^−^^3^
0 restraints	Extinction correction: *SHELXL97* (Sheldrick, 2008), Fc^*^=kFc[1+0.001xFc^2^λ^3^/sin(2θ)]^-1/4^
Primary atom site location: structure-invariant direct methods	Extinction coefficient: 0.052 (5)

### Special details

**Table:**

Geometry. All esds (except the esd in the dihedral angle between two l.s. planes) are estimated using the full covariance matrix. The cell esds are taken into account individually in the estimation of esds in distances, angles and torsion angles; correlations between esds in cell parameters are only used when they are defined by crystal symmetry. An approximate (isotropic) treatment of cell esds is used for estimating esds involving l.s. planes.
Refinement. Refinement of F^2^ against ALL reflections. The weighted R-factor wR and goodness of fit S are based on F^2^, conventional R-factors R are based on F, with F set to zero for negative F^2^. The threshold expression of F^2^ > 2sigma(F^2^) is used only for calculating R-factors(gt) etc. and is not relevant to the choice of reflections for refinement. R-factors based on F^2^ are statistically about twice as large as those based on F, and R- factors based on ALL data will be even larger.

### Fractional atomic coordinates and isotropic or equivalent isotropic displacement parameters (Å^2^)

**Table:**

	*x*	*y*	*z*	*U*_iso_*/*U*_eq_	
O1	0.44769 (19)	0.4524 (3)	0.28724 (10)	0.0314 (4)	
H1	0.4052	0.3397	0.2984	0.047*	
O2	0.3706 (2)	1.0901 (3)	0.35171 (10)	0.0335 (4)	
H2	0.4552	1.0724	0.3797	0.050*	
O3	−0.01510 (17)	0.6467 (3)	0.28917 (9)	0.0278 (4)	
H3	−0.0496	0.7427	0.3136	0.042*	
N1	0.2497 (2)	0.8010 (3)	0.23954 (10)	0.0228 (4)	
H1A	0.2320	0.6848	0.2101	0.027*	
H1B	0.3386	0.8674	0.2318	0.027*	
H1C	0.1682	0.8931	0.2297	0.027*	
C1	0.2672 (2)	0.7304 (3)	0.31806 (12)	0.0222 (5)	
C2	0.1335 (3)	0.5753 (4)	0.32623 (13)	0.0262 (5)	
H2A	0.1569	0.4334	0.3069	0.031*	
H2B	0.1280	0.5578	0.3785	0.031*	
C3	0.2617 (3)	0.9309 (4)	0.36617 (13)	0.0283 (5)	
H3A	0.1551	0.9919	0.3572	0.034*	
H3B	0.2854	0.8888	0.4179	0.034*	
C4	0.4261 (3)	0.6174 (4)	0.33775 (13)	0.0274 (5)	
H4A	0.5105	0.7245	0.3390	0.033*	
H4B	0.4340	0.5544	0.3869	0.033*	
F1	0.9653 (4)	0.7549 (5)	0.4913 (2)	0.1536 (18)	
F2	0.8144 (3)	0.5578 (3)	0.42180 (12)	0.0720 (7)	
F3	0.7347 (5)	0.7020 (5)	0.50960 (16)	0.1421 (17)	
O4	0.8379 (2)	0.9534 (3)	0.35771 (10)	0.0412 (5)	
O5	0.6631 (2)	1.0433 (4)	0.42917 (12)	0.0490 (6)	
C5	0.8217 (4)	0.7380 (5)	0.45986 (15)	0.0476 (8)	
C6	0.7677 (3)	0.9331 (4)	0.41107 (13)	0.0305 (5)	

### Atomic displacement parameters (Å^2^)

**Table:**

	*U*^11^	*U*^22^	*U*^33^	*U*^12^	*U*^13^	*U*^23^
O1	0.0273 (8)	0.0234 (8)	0.0455 (10)	0.0040 (7)	0.0114 (7)	−0.0019 (7)
O2	0.0306 (9)	0.0218 (8)	0.0464 (10)	−0.0048 (7)	0.0010 (7)	−0.0019 (7)
O3	0.0198 (8)	0.0282 (8)	0.0354 (9)	−0.0011 (6)	0.0050 (6)	−0.0026 (7)
N1	0.0198 (9)	0.0204 (9)	0.0285 (10)	−0.0003 (7)	0.0049 (7)	−0.0001 (7)
C1	0.0205 (10)	0.0197 (10)	0.0265 (11)	0.0002 (8)	0.0039 (8)	−0.0004 (8)
C2	0.0219 (10)	0.0230 (11)	0.0342 (12)	−0.0021 (9)	0.0055 (9)	0.0031 (9)
C3	0.0304 (11)	0.0226 (11)	0.0324 (12)	−0.0016 (9)	0.0063 (9)	−0.0033 (9)
C4	0.0225 (10)	0.0233 (11)	0.0353 (12)	0.0018 (9)	0.0011 (9)	0.0000 (9)
F1	0.140 (3)	0.0813 (19)	0.190 (3)	−0.0144 (18)	−0.122 (3)	0.057 (2)
F2	0.1160 (18)	0.0335 (10)	0.0696 (13)	0.0116 (11)	0.0240 (12)	0.0072 (9)
F3	0.260 (5)	0.099 (2)	0.099 (2)	0.064 (3)	0.124 (3)	0.0499 (17)
O4	0.0514 (11)	0.0379 (10)	0.0361 (10)	0.0140 (9)	0.0126 (8)	0.0091 (8)
O5	0.0337 (10)	0.0566 (13)	0.0558 (13)	0.0119 (9)	0.0047 (9)	−0.0129 (10)
C5	0.072 (2)	0.0389 (16)	0.0323 (14)	0.0066 (15)	0.0093 (14)	0.0041 (12)
C6	0.0277 (11)	0.0311 (13)	0.0311 (12)	0.0014 (10)	−0.0006 (9)	−0.0031 (10)

### Geometric parameters (Å, °)

**Table:**

O1—C4	1.400 (3)	C2—H2A	0.9700
O1—H1	0.8197	C2—H2B	0.9700
O2—C3	1.400 (3)	C3—H3A	0.9700
O2—H2	0.8202	C3—H3B	0.9700
O3—C2	1.405 (3)	C4—H4A	0.9700
O3—H3	0.8207	C4—H4B	0.9700
N1—C1	1.483 (3)	F1—C5	1.268 (4)
N1—H1A	0.8904	F2—C5	1.300 (4)
N1—H1B	0.8906	F3—C5	1.282 (4)
N1—H1C	0.8895	O4—C6	1.230 (3)
C1—C2	1.508 (3)	O5—C6	1.206 (3)
C1—C4	1.510 (3)	C5—C6	1.517 (4)
C1—C3	1.515 (3)		
			
C4—O1—H1	109.4	O2—C3—C1	111.74 (19)
C3—O2—H2	109.4	O2—C3—H3A	109.3
C2—O3—H3	109.5	C1—C3—H3A	109.3
C1—N1—H1A	109.5	O2—C3—H3B	109.3
C1—N1—H1B	109.4	C1—C3—H3B	109.3
H1A—N1—H1B	109.4	H3A—C3—H3B	107.9
C1—N1—H1C	109.5	O1—C4—C1	112.42 (18)
H1A—N1—H1C	109.5	O1—C4—H4A	109.1
H1B—N1—H1C	109.5	C1—C4—H4A	109.1
N1—C1—C2	108.68 (18)	O1—C4—H4B	109.1
N1—C1—C4	108.04 (18)	C1—C4—H4B	109.1
C2—C1—C4	110.46 (18)	H4A—C4—H4B	107.9
N1—C1—C3	108.54 (18)	F1—C5—F3	108.6 (4)
C2—C1—C3	110.94 (18)	F1—C5—F2	105.7 (3)
C4—C1—C3	110.10 (18)	F3—C5—F2	104.5 (3)
O3—C2—C1	113.05 (18)	F1—C5—C6	112.3 (3)
O3—C2—H2A	109.0	F3—C5—C6	113.4 (3)
C1—C2—H2A	109.0	F2—C5—C6	111.7 (2)
O3—C2—H2B	109.0	O5—C6—O4	129.6 (3)
C1—C2—H2B	109.0	O5—C6—C5	116.5 (2)
H2A—C2—H2B	107.8	O4—C6—C5	113.9 (2)
			
N1—C1—C2—O3	−44.3 (2)	C3—C1—C4—O1	−170.24 (18)
C4—C1—C2—O3	−162.62 (19)	F1—C5—C6—O5	−115.9 (4)
C3—C1—C2—O3	75.0 (2)	F3—C5—C6—O5	7.7 (4)
N1—C1—C3—O2	−52.4 (2)	F2—C5—C6—O5	125.5 (3)
C2—C1—C3—O2	−171.71 (19)	F1—C5—C6—O4	64.8 (4)
C4—C1—C3—O2	65.7 (2)	F3—C5—C6—O4	−171.6 (3)
N1—C1—C4—O1	−51.9 (2)	F2—C5—C6—O4	−53.8 (4)
C2—C1—C4—O1	66.9 (2)		

### Hydrogen-bond geometry (Å, °)

**Table:**

*D*—H···*A*	*D*—H	H···*A*	*D*···*A*	*D*—H···*A*
O1—H1···O2^i^	0.82	1.86	2.644 (2)	159.
O2—H2···O5	0.82	1.86	2.673 (3)	170.
O3—H3···O4^ii^	0.82	1.87	2.677 (3)	170.
N1—H1A···O4^iii^	0.89	1.91	2.795 (3)	171.
N1—H1B···O1^iv^	0.89	1.98	2.854 (2)	168.
N1—H1C···O3^v^	0.89	2.02	2.899 (2)	169.

Symmetry codes: (i) *x*, *y*−1, *z*; (ii) *x*−1, *y*, *z*; (iii) −*x*+1, *y*−1/2, −*z*+1/2; (iv) −*x*+1, *y*+1/2, −*z*+1/2; (v) −*x*, *y*+1/2, −*z*+1/2.
